# Brain injury in COVID-19 is associated with dysregulated innate and adaptive immune responses

**DOI:** 10.1093/brain/awac321

**Published:** 2022-09-06

**Authors:** Edward J Needham, Alexander L Ren, Richard J Digby, Emma J Norton, Soraya Ebrahimi, Joanne G Outtrim, Doris A Chatfield, Anne E Manktelow, Maya M Leibowitz, Virginia F J Newcombe, Rainer Doffinger, Gabriela Barcenas-Morales, Claudia Fonseca, Michael J Taussig, Rowan M Burnstein, Romit J Samanta, Cordelia Dunai, Nyarie Sithole, Nicholas J Ashton, Henrik Zetterberg, Magnus Gisslén, Arden Edén, Emelie Marklund, Peter J M Openshaw, Jake Dunning, Michael J Griffiths, Jonathan Cavanagh, Gerome Breen, Sarosh R Irani, Anne Elmer, Nathalie Kingston, Charlotte Summers, John R Bradley, Leonie S Taams, Benedict D Michael, Edward T Bullmore, Kenneth G C Smith, Paul A Lyons, Alasdair J Coles, David K Menon, the Cambridge NeuroCOVID Group the CITIID-NIHR COVID-19 BioResource Collaboration and Cambridge NIHR Clinical Research Facility

**Affiliations:** Department of Clinical Neurosciences, University of Cambridge, UK; Division of Anaesthesia, Department of Medicine, University of Cambridge, UK; Division of Anaesthesia, Department of Medicine, University of Cambridge, UK; Division of Anaesthesia, Department of Medicine, University of Cambridge, UK; Division of Anaesthesia, Department of Medicine, University of Cambridge, UK; Division of Anaesthesia, Department of Medicine, University of Cambridge, UK; Division of Anaesthesia, Department of Medicine, University of Cambridge, UK; Division of Anaesthesia, Department of Medicine, University of Cambridge, UK; Division of Anaesthesia, Department of Medicine, University of Cambridge, UK; Division of Anaesthesia, Department of Medicine, University of Cambridge, UK; Division of Anaesthesia, Department of Medicine, University of Cambridge, UK; Department of Clinical Biochemistry and Immunology, Addenbrooke's Hospital, Cambridge, UK; Department of Clinical Biochemistry and Immunology, Addenbrooke's Hospital, Cambridge, UK; Cambridge Protein Arrays Ltd, Babraham Research Campus, Cambridge, UK; Cambridge Protein Arrays Ltd, Babraham Research Campus, Cambridge, UK; Division of Anaesthesia, Department of Medicine, University of Cambridge, UK; Division of Anaesthesia, Department of Medicine, University of Cambridge, UK; Clinical Infection Microbiology and Neuroimmunology, Institute of Infection, Veterinary and Ecological Science, Liverpool, UK; Department of Infectious Diseases, Cambridge University NHS Hospitals Foundation Trust, Cambridge, UK; Department of Medicine, University of Cambridge, Addenbrooke's Hospital, Cambridge, UK; Cambridge Institute of Therapeutic Immunology and Infectious Disease, Jeffrey Cheah Biomedical Centre, University of Cambridge, Cambridge, UK; Department of Psychiatry and Neurochemistry, Institute of Neuroscience and Physiology, the Sahlgrenska Academy at the University of Gothenburg, Mölndal, Sweden; Department of Psychiatry and Neurochemistry, Institute of Neuroscience and Physiology, the Sahlgrenska Academy at the University of Gothenburg, Mölndal, Sweden; Clinical Neurochemistry Laboratory, Sahlgrenska University Hospital, Mölndal, Sweden; Department of Neurodegenerative Disease, UCL Institute of Neurology, Queen Square, London, UK; UK Dementia Research Institute at UCL, London, UK; Hong Kong Center for Neurodegenerative Diseases, Hong Kong, China; Department of Infectious Diseases, Institute of Biomedicine, the Sahlgrenska Academy at the University of Gothenburg, Gothenburg, Sweden; Region Västra Götaland, Sahlgrenska University Hospital, Department of Infectious Diseases, Gothenburg, Sweden; Department of Infectious Diseases, Institute of Biomedicine, the Sahlgrenska Academy at the University of Gothenburg, Gothenburg, Sweden; Region Västra Götaland, Sahlgrenska University Hospital, Department of Infectious Diseases, Gothenburg, Sweden; Department of Infectious Diseases, Institute of Biomedicine, the Sahlgrenska Academy at the University of Gothenburg, Gothenburg, Sweden; Region Västra Götaland, Sahlgrenska University Hospital, Department of Infectious Diseases, Gothenburg, Sweden; National Heart and Lung Institute, Imperial College London, UK; Pandemic Sciences Institute, Nuffield Department of Medicine, University of Oxford, Oxford, UK; Institute of Infection, Veterinary and Ecological Sciences, University of Liverpool, Liverpool, UK; Centre for Immunobiology, Institute of Infection, Immunity and Inflammation, College of Medical, Veterinary and Life Sciences, University of Glasgow, Glasgow, UK; Department of Social Genetic and Developmental Psychiatry, King's College London, London, UK; Oxford Autoimmune Neurology Group, Nuffield Department of Clinical Neurosciences, University of Oxford, Oxford, UK; Department of Neurology, Oxford University Hospitals NHS Foundation Trust, Oxford, UK; Cambridge Clinical Research Centre, NIHR Clinical Research Facility, Cambridge University Hospitals NHS Foundation Trust, Addenbrooke's Hospital, Cambridge, UK; NIHR BioResource, Cambridge University Hospitals NHS Foundation, Cambridge Biomedical Campus, Cambridge, UK; Department of Haematology, School of Clinical Medicine, University of Cambridge, Cambridge Biomedical Campus, Cambridge, UK; Division of Anaesthesia, Department of Medicine, University of Cambridge, UK; NIHR BioResource, Cambridge University Hospitals NHS Foundation, Cambridge Biomedical Campus, Cambridge, UK; Department of Medicine, University of Cambridge, Addenbrooke's Hospital, Cambridge, UK; NIHR BioResource, Cambridge University Hospitals NHS Foundation, Cambridge Biomedical Campus, Cambridge, UK; Centre for Inflammation Biology and Cancer Immunology (CIBCI) and Department Inflammation Biology, School of Immunology and Microbial Sciences, King’s College London, Guy's Campus, London, UK; Clinical Infection Microbiology and Neuroimmunology, Institute of Infection, Veterinary and Ecological Science, Liverpool, UK; Department of Psychiatry, University of Cambridge, Herchel Smith Building for Brain and Mind Sciences, Cambridge Biomedical Campus, Cambridge, UK; Department of Medicine, University of Cambridge, Addenbrooke's Hospital, Cambridge, UK; Cambridge Institute of Therapeutic Immunology and Infectious Disease, Jeffrey Cheah Biomedical Centre, University of Cambridge, Cambridge, UK; Department of Medicine, University of Cambridge, Addenbrooke's Hospital, Cambridge, UK; Cambridge Institute of Therapeutic Immunology and Infectious Disease, Jeffrey Cheah Biomedical Centre, University of Cambridge, Cambridge, UK; Department of Clinical Neurosciences, University of Cambridge, UK; Division of Anaesthesia, Department of Medicine, University of Cambridge, UK

**Keywords:** COVID-19, brain injury, neuroinflammation, autoantibodies

## Abstract

COVID-19 is associated with neurological complications including stroke, delirium and encephalitis. Furthermore, a post-viral syndrome dominated by neuropsychiatric symptoms is common, and is seemingly unrelated to COVID-19 severity. The true frequency and underlying mechanisms of neurological injury are unknown, but exaggerated host inflammatory responses appear to be a key driver of COVID-19 severity.

We investigated the dynamics of, and relationship between, serum markers of brain injury (neurofilament light [NfL], glial fibrillary acidic protein [GFAP] and total tau) and markers of dysregulated host response (autoantibody production and cytokine profiles) in 175 patients admitted with COVID-19 and 45 patients with influenza.

During hospitalisation, sera from patients with COVID-19 demonstrated elevations of NfL and GFAP in a severity-dependent manner, with evidence of ongoing active brain injury at follow-up 4 months later. These biomarkers were associated with elevations of pro-inflammatory cytokines and the presence of autoantibodies to a large number of different antigens. Autoantibodies were commonly seen against lung surfactant proteins but also brain proteins such as myelin associated glycoprotein. Commensurate findings were seen in the influenza cohort.

A distinct process characterised by elevation of serum total tau was seen in patients at follow-up, which appeared to be independent of initial disease severity and was not associated with dysregulated immune responses unlike NfL and GFAP.

These results demonstrate that brain injury is a common consequence of both COVID-19 and influenza, and is therefore likely to be a feature of severe viral infection more broadly. The brain injury occurs in the context of dysregulation of both innate and adaptive immune responses, with no single pathogenic mechanism clearly responsible

## Supplementary Material

awac321_Supplementary_Data

