# Brain injury in COVID-19 is associated with dysregulated innate and adaptive immune responses

**DOI:** 10.1093/brain/awac321

**Published:** 2022-09-06

**Authors:** Edward J Needham, Alexander L Ren, Richard J Digby, Emma J Norton, Soraya Ebrahimi, Joanne G Outtrim, Doris A Chatfield, Anne E Manktelow, Maya M Leibowitz, Virginia F J Newcombe, Rainer Doffinger, Gabriela Barcenas-Morales, Claudia Fonseca, Michael J Taussig, Rowan M Burnstein, Romit J Samanta, Cordelia Dunai, Nyarie Sithole, Nicholas J Ashton, Henrik Zetterberg, Magnus Gisslén, Arvid Edén, Emelie Marklund, Peter J M Openshaw, Jake Dunning, Michael J Griffiths, Jonathan Cavanagh, Gerome Breen, Sarosh R Irani, Anne Elmer, Nathalie Kingston, Charlotte Summers, John R Bradley, Leonie S Taams, Benedict D Michael, Edward T Bullmore, Kenneth G C Smith, Paul A Lyons, Alasdair J Coles, David K Menon, Cambridge NeuroCOVID Group, Fahim Anwar, Kieren Allinson, Junaid Bhatti, Edward T Bullmore, Dorothy A Chatfield, David Christmas, Alasdair J Coles, Jonathan P Coles, Marta Correia, Tilak Das, Paul C Fletcher, Alasdair W Jubb, Victoria C Lupson, Anne E Manktelow, David K Menon, Andrew Michell, Edward J Needham, Virginia F J Newcombe, Joanne G Outtrim, Linda Pointon, Christopher T Rodgers, James B Rowe, Catarina Rua, Nyarie Sithole, Lennart R B Spindler, Emmanuel A Stamatakis, Jonathan Taylor, Fernanda Valerio, Barry Widmer, Guy B Williams, Patrick F Chinnery, CITIID-NIHR COVID-19 BioResource Collaboration, John Allison, Gisele Alvio, Ali Ansaripour, Sharon Baker, Stephen Baker, Laura Bergamaschi, Areti Bermperi, Ariana Betancourt, Heather Biggs, Sze-How Bong, Georgie Bower, John R Bradley, Karen Brookes, Ashlea Bucke, Ben Bullman, Katherine Bunclark, Helen Butcher, Sarah Caddy, Jo Calder, Laura Caller, Laura Canna, Daniela Caputo, Matt Chandler, Yasmin Chaudhry, Patrick Chinnery, Debbie Clapham-Riley, Daniel Cooper, Chiara Cossetti, Cherry Crucusio, Isabel Cruz, Martin Curran, Jerome D Coudert, Eckart M D D De Bie, Rnalie De Jesus, Aloka De Sa, Anne-Maree Dean, Katie Dempsey, Eleanor Dewhurst, Giovanni di Stefano, Jason Domingo, Gordon Dougan, Benjamin J Dunmore, Anne Elmer, Madeline Epping, Codie Fahey, Stuart Fawke, Theresa Feltwell, Christian Fernandez, Stewart Fuller, Anita Furlong, Iliana Georgana, Anne George, Nick Gleadall, Ian G Goodfellow, Stefan Gräf, Barbara Graves, Jennifer Gray, Richard Grenfell, Ravindra K Gupta, Grant Hall, William Hamilton, Julie Harris, Sabine Hein, Christoph Hess, Sarah Hewitt, Andrew Hinch, Josh Hodgson, Myra Hosmillo, Elaine Holmes, Charlotte Houldcroft, Christopher Huang, Oisín Huhn, Kelvin Hunter, Tasmin Ivers, Aminu Jahun, Sarah Jackson, Isobel Jarvis, Emma Jones, Heather Jones, Sherly Jose, Maša Josipović, Mary Kasanicki, Jane Kennet, Fahad Khokhar, Yvonne King, Nathalie Kingston, Jenny Kourampa, Emma Le Gresley, Elisa Laurenti, Ekaterina Legchenko, Paul J Lehner, Daniel Lewis, Emily Li, Rachel Linger, Paul A Lyons, Michael Mackay, John C Marioni, Jimmy Marsden, Jennifer Martin, Cecilia Matara, Nicholas J Matheson, Caroline McMahon, Anne Meadows, Sarah Meloy, Vivien Mendoza, Luke Meredith, Nicole Mende, Federica Mescia, Alice Michael, Alexei Moulton, Rachel Michel, Lucy Mwaura, Francesca Muldoon, Francesca Nice, Criona O’Brien, Charmain Ocaya, Ciara O’Donnell, Georgina Okecha, Ommar Omarjee, Nigel Ovington, Willem H Owehand, Sofia Papadia, Roxana Paraschiv, Surendra Parmar, Ciro Pascuale, Caroline Patterson, Christopher Penkett, Marlyn Perales, Marianne Perera, Isabel Phelan, Malte Pinckert, Linda Pointon, Petra Polgarova, Gary Polwarth, Nicole Pond, Jane Price, Venkatesh Ranganath, Cherry Publico, Rebecca Rastall, Carla Ribeiro, Nathan Richoz, Veronika Romashova, Sabrina Rossi, Jane Rowlands, Valentina Ruffolo, Jennifer Sambrook, Caroline Saunders, Natalia Savinykh Yarkoni, Katherine Schon, Mayurun Selvan, Rahul Sharma, Joy Shih, Kenneth G C Smith, Sarah Spencer, Luca Stefanucci, Hannah Stark, Jonathan Stephens, Kathleen E Stirrups, Mateusz Strezlecki, Charlotte Summers, Rachel Sutcliffe, James E D Thaventhiran, Tobias Tilly, Zhen Tong, Hugo Tordesillas, Carmen Treacy, Mark Toshner, Paul Townsend, Carmen Treacy, Lori Turner, Phoebe Vargas, Bensi Vergese, Julie von Ziegenweidt, Neil Walker, Laura Watson, Jennifer Webster, Michael P Weekes, Nicola K Wilson, Jennifer Wood, Jieniean Worsley, Marta Wylot, Anna Yakovleva, Cissy Yong and Julie-Anne Zerrudo, Cambridge NIHR Clinical Research Facility, Caroline Saunders, Anne Elmer

**Affiliations:** Department of Clinical Neurosciences, University of Cambridge, UK; Division of Anaesthesia, Department of Medicine, University of Cambridge, UK; Division of Anaesthesia, Department of Medicine, University of Cambridge, UK; Division of Anaesthesia, Department of Medicine, University of Cambridge, UK; Division of Anaesthesia, Department of Medicine, University of Cambridge, UK; Division of Anaesthesia, Department of Medicine, University of Cambridge, UK; Division of Anaesthesia, Department of Medicine, University of Cambridge, UK; Division of Anaesthesia, Department of Medicine, University of Cambridge, UK; Division of Anaesthesia, Department of Medicine, University of Cambridge, UK; Division of Anaesthesia, Department of Medicine, University of Cambridge, UK; Division of Anaesthesia, Department of Medicine, University of Cambridge, UK; Department of Clinical Biochemistry and Immunology, Addenbrooke’s Hospital, Cambridge, UK; Department of Clinical Biochemistry and Immunology, Addenbrooke’s Hospital, Cambridge, UK; Cambridge Protein Arrays Ltd, Babraham Research Campus, Cambridge, UK; Cambridge Protein Arrays Ltd, Babraham Research Campus, Cambridge, UK; Division of Anaesthesia, Department of Medicine, University of Cambridge, UK; Division of Anaesthesia, Department of Medicine, University of Cambridge, UK; Clinical Infection Microbiology and Neuroimmunology, Institute of Infection, Veterinary and Ecological Science, Liverpool, UK; Department of Infectious Diseases, Cambridge University NHS Hospitals Foundation Trust, Cambridge, UK; Department of Medicine, University of Cambridge, Addenbrooke's Hospital, Cambridge, UK; Jeffrey Cheah Biomedical Centre, Cambridge Institute of Therapeutic Immunology and Infectious Disease, University of Cambridge, Cambridge, UK; Department of Psychiatry and Neurochemistry, Institute of Neuroscience and Physiology, the Sahlgrenska Academy at the University of Gothenburg, Mölndal, Sweden; Department of Psychiatry and Neurochemistry, Institute of Neuroscience and Physiology, the Sahlgrenska Academy at the University of Gothenburg, Mölndal, Sweden; Clinical Neurochemistry Laboratory, Sahlgrenska University Hospital, Mölndal, Sweden; Department of Neurodegenerative Disease, UCL Institute of Neurology, Queen Square, London, UK; UK Dementia Research Institute at UCL, London, UK; Hong Kong Center for Neurodegenerative Diseases, Hong Kong, China; Department of Infectious Diseases, Institute of Biomedicine, the Sahlgrenska Academy at the University of Gothenburg, Gothenburg, Sweden; Department of Infectious Diseases, Region Västra Götaland, Sahlgrenska University Hospital, Gothenburg, Sweden; Department of Infectious Diseases, Institute of Biomedicine, the Sahlgrenska Academy at the University of Gothenburg, Gothenburg, Sweden; Department of Infectious Diseases, Region Västra Götaland, Sahlgrenska University Hospital, Gothenburg, Sweden; Department of Infectious Diseases, Institute of Biomedicine, the Sahlgrenska Academy at the University of Gothenburg, Gothenburg, Sweden; Department of Infectious Diseases, Region Västra Götaland, Sahlgrenska University Hospital, Gothenburg, Sweden; National Heart and Lung Institute, Imperial College London, London, UK; Nuffield Department of Medicine, Pandemic Sciences Institute, University of Oxford, Oxford, UK; Institute of Infection, Veterinary and Ecological Sciences, University of Liverpool, Liverpool, UK; Centre for Immunobiology, Institute of Infection, Immunity and Inflammation, College of Medical, Veterinary and Life Sciences, University of Glasgow, Glasgow, UK; Department of Social Genetic and Developmental Psychiatry, King’s College London, London, UK; Oxford Autoimmune Neurology Group, Nuffield Department of Clinical Neurosciences, University of Oxford, Oxford, UK; Department of Neurology, Oxford University Hospitals NHS Foundation Trust, Oxford, UK; Cambridge Clinical Research Centre, NIHR Clinical Research Facility, Cambridge University Hospitals NHS Foundation Trust, Addenbrooke's Hospital, Cambridge, UK; NIHR BioResource, Cambridge University Hospitals NHS Foundation, Cambridge Biomedical Campus, Cambridge, UK; Department of Haematology, School of Clinical Medicine, University of Cambridge, Cambridge Biomedical Campus, Cambridge, UK; Division of Anaesthesia, Department of Medicine, University of Cambridge, UK; NIHR BioResource, Cambridge University Hospitals NHS Foundation, Cambridge Biomedical Campus, Cambridge, UK; Department of Medicine, University of Cambridge, Addenbrooke's Hospital, Cambridge, UK; NIHR BioResource, Cambridge University Hospitals NHS Foundation, Cambridge Biomedical Campus, Cambridge, UK; Centre for Inflammation Biology and Cancer Immunology (CIBCI) and Department Inflammation Biology, School of Immunology and Microbial Sciences, King’s College London, Guy's Campus, London, UK; Clinical Infection Microbiology and Neuroimmunology, Institute of Infection, Veterinary and Ecological Science, Liverpool, UK; Department of Psychiatry, University of Cambridge, Herchel Smith Building for Brain and Mind Sciences, Cambridge Biomedical Campus, Cambridge, UK; Department of Medicine, University of Cambridge, Addenbrooke's Hospital, Cambridge, UK; Jeffrey Cheah Biomedical Centre, Cambridge Institute of Therapeutic Immunology and Infectious Disease, University of Cambridge, Cambridge, UK; Department of Medicine, University of Cambridge, Addenbrooke's Hospital, Cambridge, UK; Jeffrey Cheah Biomedical Centre, Cambridge Institute of Therapeutic Immunology and Infectious Disease, University of Cambridge, Cambridge, UK; Department of Clinical Neurosciences, University of Cambridge, UK; Division of Anaesthesia, Department of Medicine, University of Cambridge, UK

**Keywords:** COVID-19, brain injury, neuroinflammation, autoantibodies

## Abstract

COVID-19 is associated with neurological complications including stroke, delirium and encephalitis. Furthermore, a post-viral syndrome dominated by neuropsychiatric symptoms is common, and is seemingly unrelated to COVID-19 severity. The true frequency and underlying mechanisms of neurological injury are unknown, but exaggerated host inflammatory responses appear to be a key driver of COVID-19 severity.

We investigated the dynamics of, and relationship between, serum markers of brain injury [neurofilament light (NfL), glial fibrillary acidic protein (GFAP) and total tau] and markers of dysregulated host response (autoantibody production and cytokine profiles) in 175 patients admitted with COVID-19 and 45 patients with influenza.

During hospitalization, sera from patients with COVID-19 demonstrated elevations of NfL and GFAP in a severity-dependent manner, with evidence of ongoing active brain injury at follow-up 4 months later. These biomarkers were associated with elevations of pro-inflammatory cytokines and the presence of autoantibodies to a large number of different antigens. Autoantibodies were commonly seen against lung surfactant proteins but also brain proteins such as myelin associated glycoprotein. Commensurate findings were seen in the influenza cohort.

A distinct process characterized by elevation of serum total tau was seen in patients at follow-up, which appeared to be independent of initial disease severity and was not associated with dysregulated immune responses unlike NfL and GFAP.

These results demonstrate that brain injury is a common consequence of both COVID-19 and influenza, and is therefore likely to be a feature of severe viral infection more broadly. The brain injury occurs in the context of dysregulation of both innate and adaptive immune responses, with no single pathogenic mechanism clearly responsible.

## Teaser

Needham *et al.* reveal elevations in blood biomarkers of brain injury in patients hospitalised with COVID-19. The changes, which were severity-dependent, were associated with dysregulated immune responses including increases in pro-inflammatory cytokines and autoantibodies. Ongoing active brain injury could still be seen months after infection.

**See Bauer and Reindl (https://doi.org/10.1093/brain/awac368) for a scientific commentary on this article.**

## Introduction

COVID-19 has been associated with several neurological complications including stroke and immune-mediated disorders such as Guillain-Barré syndrome and autoimmune encephalitis.^1^ Furthermore, up to a third of infected individuals experience a protracted post-viral syndrome following COVID-19, which is likely of CNS origin given the dominance of neuropsychiatric symptoms such as fatigue and subjective cognitive difficulties.^2–4^ While the occurrence of physical brain injury is overt in some COVID-19-associated neurological syndromes such as stroke and encephalitis, a number of studies have suggested that brain injury can occur in the context of COVID-19 even in the absence of a clear concomitant neurological diagnosis. However, the mechanism that might drive this process requires further attention. ^5–16^ In COVID-19 disease, exaggerated host inflammatory responses appear to be a key driver of severe disease, and the most effective established therapies for systemic COVID-19 aim to attenuate this response.^17,18^ Initial attention focused on the innate immune system as a key driver, but emerging evidence also suggests a role for dysregulated adaptive immune responses.^19^ This combined maladaptive response is reminiscent of that seen in a spectrum of immune-mediated diseases—which extend from autoinflammatory to autoimmune in nature.^20^ Well established, clinically-relevant neuronal surface or intracellular autoantibodies have only rarely been found in the serum of patients with COVID-19,^21,22^ but indirect immunofluorescence studies on brain sections suggest other autoantibodies may be relevant.^21^ Standard autoantibody assays are optimized to detect specific, high-affinity antibodies, but a significant proportion of the immunoglobulin repertoire consists of low-affinity autoantibodies, such as natural autoantibodies, which have less well-defined biological roles in infection, homeostasis and autoimmunity.^23^

Here, we investigated markers of a dysregulated host immune response, including surrogates of maladaptive innate (proinflammatory cytokines) and adaptive (autoantibodies) inflammation, and how they correlated with biomarkers of brain injury.

## Materials and methods

### Study populations

Patients admitted to Cambridge University Hospital, UK with PCR-proven COVID-19 were identified between March 2020 and March 2021. Providing research personnel were available, all patients admitted to Cambridge were approached for consent, either in the acute phase, or at follow-up visit. The cohort of patients recruited from Cambridge were supplemented by a convenience sample of PCR-proven COVID-19 patients from Sahlgrenska University Hospital, Sweden (February–March 2020); previously included in a prospective sampling study.^24^ Written consent was gained from either patients themselves, or from their legal representatives where they lacked capacity to consent. Where written consent could not be gained due to restrictions on hospital visiting, legal representatives were consulted by telephone. This study was approved by the Swedish Ethical Review Authority (2020–01771) and the East of England—Cambridge Central Research Ethics Committee (17/EE/0025); via the Cambridge Biomedical Research Centre. Healthy controls were recruited through the Cambridge Biomedical Research Centre (prior to the COVID-19 pandemic) and all provided written consent (17/EE/0025). Data from a small positive control group consisting of patients with acute severe traumatic brain injury were included as a reference for the magnitude of brain injury biomarker elevations (REC 97/290). Stored plasma and clinical data from patients with influenza infection who were recruited to the MOSAIC trial ^25^ (REC 09/H0709/52, 09/MRE00/67) were used as a further control cohort.

### Procedures

Serum samples were collected at up to three time points from admission [acute (0–14 days), subacute (15–70 days) and convalescent (at outpatient follow-up; >80 days)]. The samples were aliquoted, labelled with pseudoanonymized identifiers, and frozen immediately at −70°C. Samples from Sweden were then shipped on dry ice to the University of Cambridge.

### Demographic and clinical information

Demographic, clinical and laboratory information was recorded by the clinical team at the time of admission; Short Form Health Survey 36 (SF36)^26^ was completed in patients recruited to Cambridge University Hospital who returned for follow-up after their attendance to hospital. Patients with COVID-19 or influenza were stratified into three groups of severity based on the treatment needed in the acute phase (mild: no supplemental oxygen was required; moderate: supplemental oxygen was required; severe: invasive mechanical ventilation was required).

### Brain injury biomarker measurement

Neurofilament light (NfL), glial fibrillary acidic protein (GFAP), total tau and ubiquitin C-terminal hydrolase L1 concentrations were quantified in serum (COVID-19 patients and relevant control group) or plasma (influenza patients and relevant control group) at the University of Cambridge using the Neurology 4-PLEX A assay run on an HD-X Analyser (Quanterix). As per previous experience, UCH-L1 levels were predominantly below the functional lower level of quantification (with only 12% all samples demonstrating concentrations above this level), with high coefficients of variance between replicates, and therefore were excluded from analysis (data are displayed for completeness in Supplementary Fig. 1). Five serum samples taken from patients within 3 days of severe traumatic brain injury were also assayed to provide a frame of reference for magnitude of changes seen.

### Protein microarray autoantibody profiling

Autoantibody screening was performed using a custom CNS protein microarray based on the HuProt™ (version 4.0) platform.^27,28^ The microarray was devised in collaboration with Cambridge Protein Arrays Ltd. and CDI laboratories to detect autoantibodies predominantly directed against CNS antigens (*n* = 51), but also to a number of blood–brain barrier (*n* = 5) and other tissue-specific (*n* = 94, covering organ systems including lung, heart and coagulation) antigens, as well as spike and nucleocapsid antigens (full antigen list detailed in Supplementary Fig. 2). The microarrays consist of a glass microscope slide with a thin nitrocellulose coating, printed with quadruplicate spots of recombinant yeast-expressed whole proteins. Each slide accommodates up to 12 individual serum samples. Samples from healthy controls and patients with COVID-19 were randomly distributed across the slides to mitigate against experimental variation.

The slides were blocked in 2% bovine serum albumin (BSA)/0.1% PBS-Tween overnight at 4°C, washed, and then incubated with 200 μl of 1:1000 diluted serum at room temperature for 2 h. The slides were washed again, incubated at room temperature for 2 h with fluorophore-conjugated goat anti-human IgM-μ chain-Alexa488 (Invitrogen, Cat. No. A21215) and goat anti-human IgG-Fc-DyLight550 (Invitrogen Cat. No. SA5–10135) secondary antibodies, washed, and then scanned using a Tecan LS400 scanner and GenePix Pro v4 software, with the output being median fluorescence value of the quadruplicate spots for each protein.

### Cytokine profiling

Serum concentrations of TNFα, IL-1β, IL-6, IL-10 and IFN-γ were quantified using by multiplexed particle-based flow cytometry on a Luminex 200 analyser using xPonent Software (R&D Systems/Luminex) according to manufacturer’s recommendations. The population reference ranges derived for clinical use with this assay were utilized. Sensitivities/minimum detectable doses as indicated by the manufacturer are: IFN-γ (0.04 pg/ml); IL-1β (0.08 pg/ml); IL-6 (0.14 pg/ml); IL10 (0.21 pg/ml); TNFα (0.29 pg/ml).

Plasma concentrations of cytokines in the influenza cohort were determined using the MSD SECTOR instrument, as described in the MOSAIC study.^25^

### Statistical analysis

Continuous descriptive data are presented using median and interquartile range (IQR), and categorical variables using number and percentage. Unpaired two-group comparisons were assessed using Mann-Whitney U-tests, paired two-group comparisons with Wilcoxon Matched-Pairs Signed Rank tests and categorical comparisons with the Chi-squared statistic. Multiple *t*-tests were used to generate volcano-plots, with a false-discovery rate (FDR) set to 1%. Comparisons between more than two groups were undertaken using Kruskal-Wallis test with *post hoc* Dunn’s multiple comparison test. Correlations between continuous variables were assessed using Spearman’s rank correlation co-efficient, and where multiple correlations were assessed within an experiment, Bonferroni correction was used to determine the appropriate level of significance. Principal component analysis was used as a dimension reduction technique to identify inflammatory cytokine profiles. All analyses were performed using GraphPad Prism Version 9.2.0.

#### Protein microarray data analysis

As previously described,^28^ antibody binding was determined by measuring the median fluorescence intensity (MFI) of the four quadruplicate spots of each antigen; this value was then normalized by dividing it by the median MFI value of all antigens for that sample. These normalized values were then transformed into Z-scores based on the distribution derived for each antigen from the healthy control cohort. A positive autoantibody ‘hit’ was defined as an antigen where Z ≥ 3.

### Data availability

All data are available from the corresponding author on request.

## Results

### Study populations

For brain injury biomarker analysis, 250 samples [from 175 patients (122 from Cambridge University Hospital, Cambridge, UK and 53 from Sahlgrenska University Hospital, Gothenburg, Sweden) at up to three time points], and control samples from 59 age-matched healthy individuals and 45 patients admitted with influenza were obtained (all prior to the pandemic). The 122 patients from Cambridge represented ∼7% of a total of 1666 patients admitted over the recruitment period, and the 53 patients from Gothenburg represented ∼39% of a total of 137 patients admitted over the recruitment period. Comparisons of the study populations with the overall admitted populations are shown in Supplementary Fig. 3. Overall, there was no difference in age between patients and healthy controls [51 (35–61) versus 50 (32–62)], but a larger proportion of males in the patient group [93 (53%) versus 21 (35%); *P* = 0.02]. Of the COVID-19 patients, 70 (40%) had mild disease, 72 (41%) moderate disease and 33 (19%) severe disease. The median (IQR) timings of the samples post-admission were: acute = 7 (3–10) days, subacute = 31 (26–35) days, and convalescent = 122 (109–136). A subset of these patients underwent autoantibody (*n* = 122) and cytokine profiling (*n* = 82). Descriptions of all cohorts and samples are shown in Supplementary Fig. 3.

### Acute brain injury increases with COVID-19 severity, but late tau elevation is severity-independent

**Figure 1:**
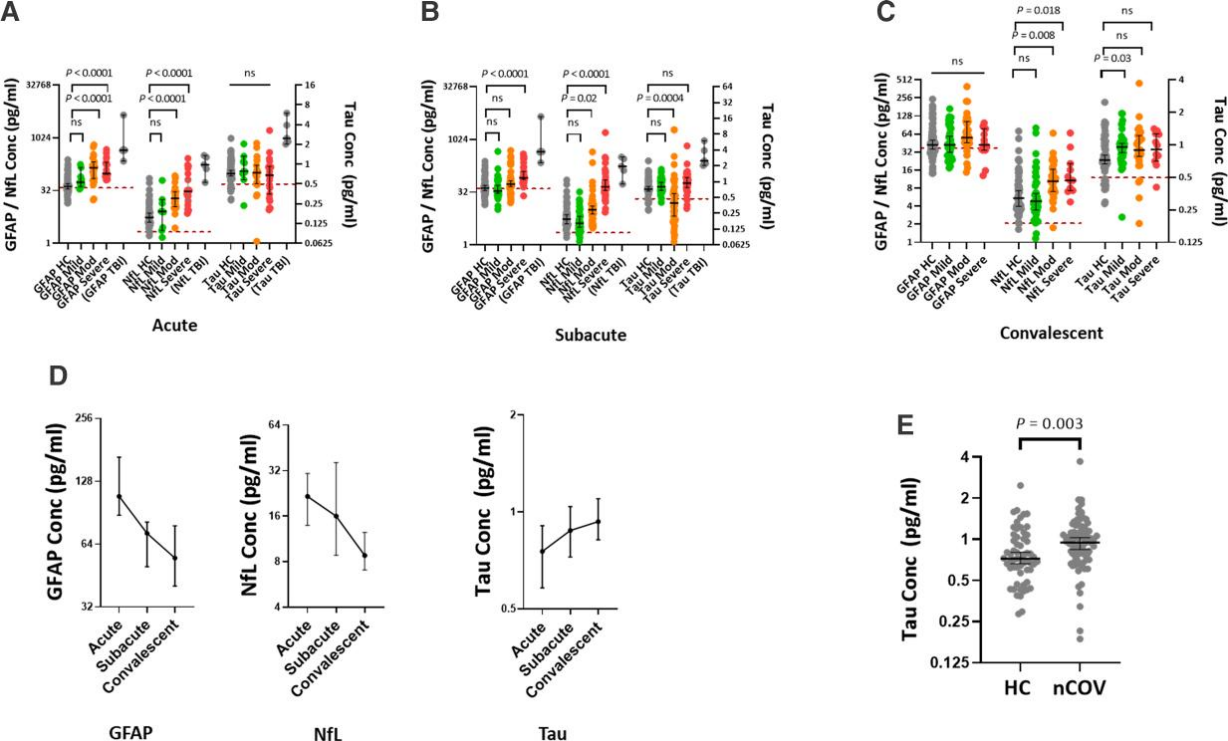
**Serum brain injury biomarker concentrations in patients with COVID-19.** (**A**–**C**) Dot plots showing the effect of COVID-19 disease severity on brain injury biomarkers at the acute, subacute and convalescent time points; representative levels from five patients with acute severe traumatic brain injury (TBI) included as a reference for magnitude of elevation. Maroon dashed line denotes the functional lower limit of quantification. (**D**) Temporal changes in serum GFAP, NfL and tau concentrations. (**E**) Elevated serum total tau concentrations at the convalescent time point in COVID-19. HC = healthy controls; nCOV = COVID-19; TBI = traumatic brain injury; CNS = central nervous system complication; PNS = peripheral nervous system complication. Multiple group comparisons are by Kruskal-Wallis test with *post hoc* Dunn’s multiple comparison test; two-group unpaired comparisons are by Mann-Whitney U-test, and paired by Wilcoxon matched-pairs signed rank test; correlations are by Spearman’s rank.

To quantify the magnitude of brain injury, we measured serum concentrations of blood brain-injury biomarkers using the Quanterix Simoa Neuro 4-PLEX B assay; concentrations of NfL, GFAP and total tau above the functional lower limit of quantification of the assays were detectable in most health control serum samples (NfL 99%, GFAP 69% and total tau 51%) and COVID-19 serum samples (NfL 97%, GFAP 73% and total tau 77%). In patients with COVID-19, serum concentrations of NfL and GFAP rose in a severity-dependant manner at both the acute and subacute time points, with a magnitude equal to the levels seen following severe traumatic brain injury in some patients; there was no consistent difference between serum total tau concentrations between patients and controls (Fig. 1A and B and Supplementary Table 1).

The temporal dynamics, in 67 patients who provided longitudinal samples, showed that both GFAP and NfL tended to fall with time, although NfL rose in some patients between the acute and subacute time points, presumably as a result of its longer half-life (Fig. 1D and Supplementary Fig. 4A). Unusually, serum total tau concentrations were significantly higher than controls at the convalescent time point [0.95 (0.75–1.15) versus 0.72 (0.60–1.04) pg/ml, *P* = 0.003; Fig. 1D and E].

At the convalescent time point, serum GFAP concentrations were no higher than controls irrespective of disease severity, but serum NfL concentrations persisted at levels that were higher in patients who had developed moderate and severe COVID-19 compared with controls (Fig. 1C and Supplementary Table 1). The elevation of serum total tau concentration did not vary with severity, and indeed after correction for multiple comparisons only patients who had developed mild disease remained significantly higher than controls (Fig. 1C and Supplementary Table 1). Convalescent levels of both NfL and GFAP concentrations correlated with paired samples taken at the 15–42 day time point (ρ = 0.69, *P* = 0.0008 and ρ = 0.82, *P* < 0.0001, respectively), but total tau did not (ρ = 0.27, *P* = 0.02), suggesting that the residual elevations of NfL and GFAP are reflective of events occurring during the acute illness, whereas the subsequent elevation of total tau appears to be independent from any acute effects.

Given the multiple comparisons above, we performed a sensitivity analysis using a mixed effects model which confirmed that both severity and time point significantly affected both GFAP (*P* = 0.0017 and *P* < 0.0001) and NfL (*P* = 0.003 and *P* < 0.0001), but not total tau (*P* = 0.81 and *P* = 0.71) concentrations in the serum of COVID-19 patients. There was no significant interaction between severity and time point for either GFAP (*P* = 0.06) or NfL (*P* = 0.13).

To explore the relationship between elevations of convalescent brain injury biomarkers and clinical outcomes, we studied correlations with the eight components of the SF-36. High convalescent serum NfL concentrations appeared to correlate most strongly with worse scores [notably: physical functioning (ρ = −0.52, *P* = 0.03), general health (ρ = −0.48, *P* = 0.05) and role functioning–emotional (ρ = −0.53, *P* = 0.02)]. The relationship between serum total tau concentrations and SF-36 domains, however, was very different, with higher concentrations seemingly associating with better scores, particularly in the emotional components [emotional wellbeing (ρ = 0.56, *P* = 0.02) and energy/vitality (ρ = 0.56, *P* = 0.02); Supplementary Fig. 4B]. However, none of the above comparisons withstood adjustments for multiple comparisons.

While the number of patients in this cohort with specific neurological syndromic diagnoses were small (mononeuritis multiplex *n* = 3, opsoclonus myoclonus *n* = 1, and peripheral neuropathy with concurrent encephalopathy *n* = 1), these patients did not appear to have higher brain injury biomarker levels, with only one patient showing biomarker levels an order of magnitude higher than the non-neurological patients (Supplementary Fig. 4C).

To determine whether elevations in brain-injury biomarkers were specific to COVID-19, we measured them in the subacute plasma of 45 patients admitted with influenza [age: 44 (30–50) years; sex: 51% male; sample time point: 34 (29–41) days post-admission; severity: mild 49%, moderate 33%, severe 18%] and 16 healthy controls. Whilst the absolute concentrations are not directly comparable with the COVID-19 cohort (as the samples were plasma rather than serum), GFAP and NfL were elevated in patients with severe disease to a similar magnitude as the COVID-19 cohort (Supplementary Fig. 4D).

### Diverse autoantibodies are seen in COVID-19 and associate with proinflammatory cytokine profiles

**Figure 2:**
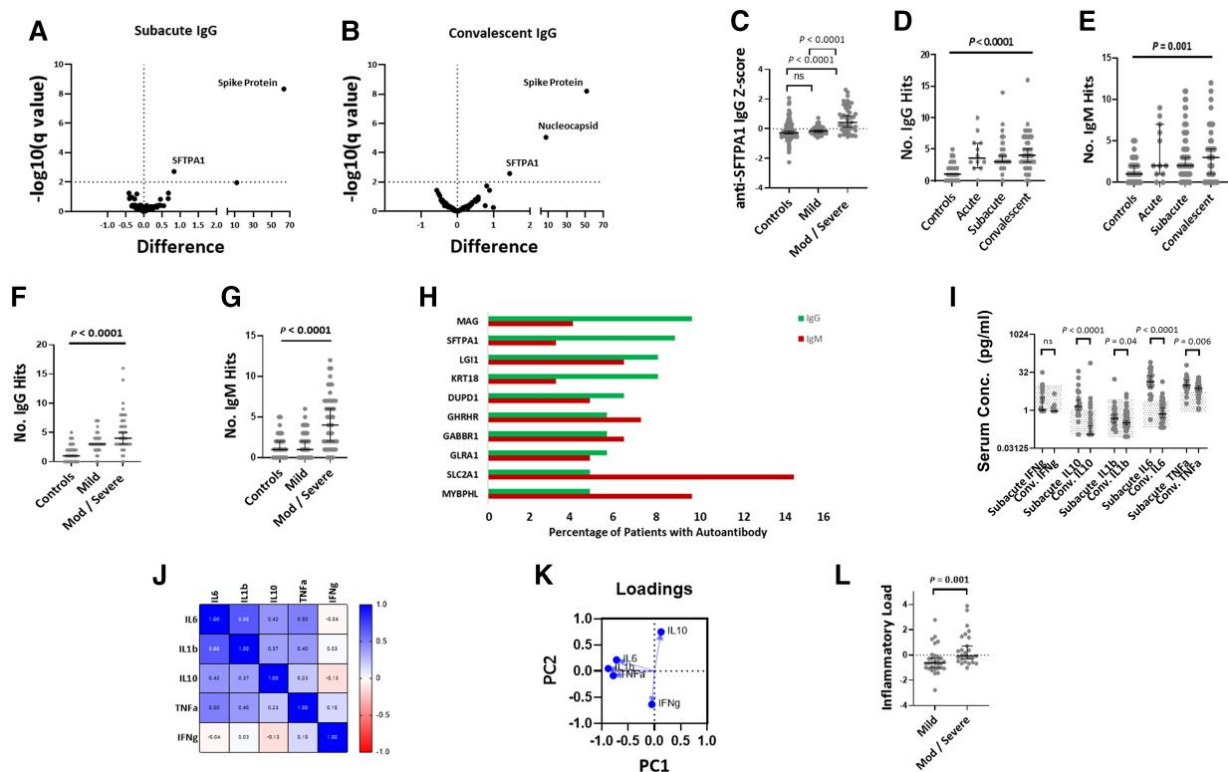
**Immune profiling in COVID-19.** (**A** and **B**) Volcano plots of groupwise comparisons in autoantibody profiles between COVID-19 patients and controls. (**C**) Relationship between disease severity and anti-SFTPA1 IgG autoantibodies. (**D** and **E**) Temporal profiles of IgG and IgM autoantibody responses. (**F** and **G**) Effect of disease severity on number of IgG and IgM autoantibody ‘hits’. (**H**) Top 10 most frequently detected autoantibodies across all samples. (**I**) Comparison of cytokine profiles at the subacute and convalescent time points, with normal range shown by hatching. (**J**) Correlation matrix between measured subacute cytokines. (**K**) Loadings plot from principal component analysis demonstrating the contributions of proinflammatory cytokines to PC1. (**L**) Comparison in subacute proinflammatory cytokine response between mild and moderate/severe disease (‘Inflammatory Load’ = the inverse of cytokine PC1). Volcano plots use multiple Mann-Whitney U-tests with an FDR rate set to 1%; multiple group comparisons are by Kruskal-Wallis test with *post hoc* Dunn’s multiple comparison test; two-group unpaired comparisons are by Mann-Whitney U-test, correlation matrix is by Spearman’s rank.

To assess whether autoantibodies were detected in patients with COVID-19, we screened serum for autoantibodies using a custom-designed protein microarray (see ‘Methods and methods’ section for details).^28^ The data were first assessed for any group-wise differences in reactivity to self-antigens between patients with COVID-19 and controls; volcano plots showed that not only did COVID-19 patients demonstrate clear IgG reactivity to SARS-CoV-2 spike protein and nucleocapsid, but also to surfactant protein A (SFTPA1), a lung surfactant protein, mutations of which result in pulmonary fibrosis (Fig. 2A).^29^ This increased reactivity was seen in both subacute and convalescent samples (Fig. 2B); reactivity to SFTPA1 in the subacute samples was stronger in patients with moderate and severe disease than in either those with mild disease or healthy controls (Fig. 2C). The presence of this autoantibody has not been previously described in COVID-19; furthermore, we have not detected it in cohorts of patients with traumatic brain injury (unpublished data), suggesting that it is not a common finding in critically ill patients more generally. No increased IgM reactivities were seen to any antigen in subacute COVID-19 samples compared with controls, but there was higher IgM reactivity to both spike protein and HLA-DRA in the convalescent samples.

While the group level comparisons provided information about pervasive autoantibody responses that were common across patients, this approach was less useful in identifying autoantibody responses which were found in a minority of patients but were still biologically interesting. Autoantibody profiles of the groups were therefore compared by assessing the number and targets of positive autoantibody hits to specific target antigens. COVID-19 patients had higher numbers of both IgG and IgM autoantibody hits than healthy controls, which peaked at the subacute time point, but remained elevated in the convalescent samples (Fig. 2D and E). Patients with moderate or severe disease had higher numbers of autoantibody hits than those with mild disease at the subacute time point (Fig. 2F and G), and the number of IgM and IgG autoantibodies in an individual were related (ρ = 0.32, *P* = 0.01).

Autoantibodies to many different antigens were seen, but some were seen more frequently (Fig. 2H). Anti-myelin associated glycoprotein (MAG) was the most commonly detected IgG autoantibody, seen in 9.6% COVID-19 samples but not seen in any healthy controls, followed by surfactant protein A (SFTPA1), which was detected in 8.8% patients, and again not seen in healthy controls (frequency of positive autoantibody hits in control and COVID-19 cohorts shown in Supplementary Table 2). Most of these responses were of low signal strength, but very high strength signal was seen in those demonstrating anti-interferon alpha antibodies (Supplementary Fig. 5). No specifically characteristic autoantibody was seen in the five patients with syndromic neurological diagnoses.

Serum cytokine profiling was undertaken by Luminex®. Elevations in serum cytokine concentrations were seen in the subacute samples, particularly IL-6, TNFα and IL-10, but many patients demonstrated concentrations persisting above the normal range in the convalescent samples. (Fig. 2I). There was substantial covariance between all cytokines other than interferon gamma (Fig. 2J), but principal component analysis demonstrated three canonical pro-inflammatory cytokines (IL-1β, IL-6 and TNFα) driving PC1 (Fig. 2K). Given the negative direction of the pro-inflammatory eigenvector of PC1, a ‘pro-inflammatory load’ score was generated by simply inverting the PC1 eigenvalue to aid clarity of communication (so that higher concentrations of pro-inflammatory cytokines were represented by a higher ‘pro-inflammatory load’ score). Patients with moderate and severe disease demonstrated higher concentrations of proinflammatory cytokines (Fig. 2L). The number of both IgG and IgM hits correlated with an elevated proinflammatory cytokine response (pro-inflammatory load score versus IgG: ρ = 0.33, *P* = 0.01, versus IgM: ρ = 0.30, *P* = 0.02).

### Magnitude of autoantibody and pro-inflammatory cytokine response correlate with brain injury

**Figure 3:**
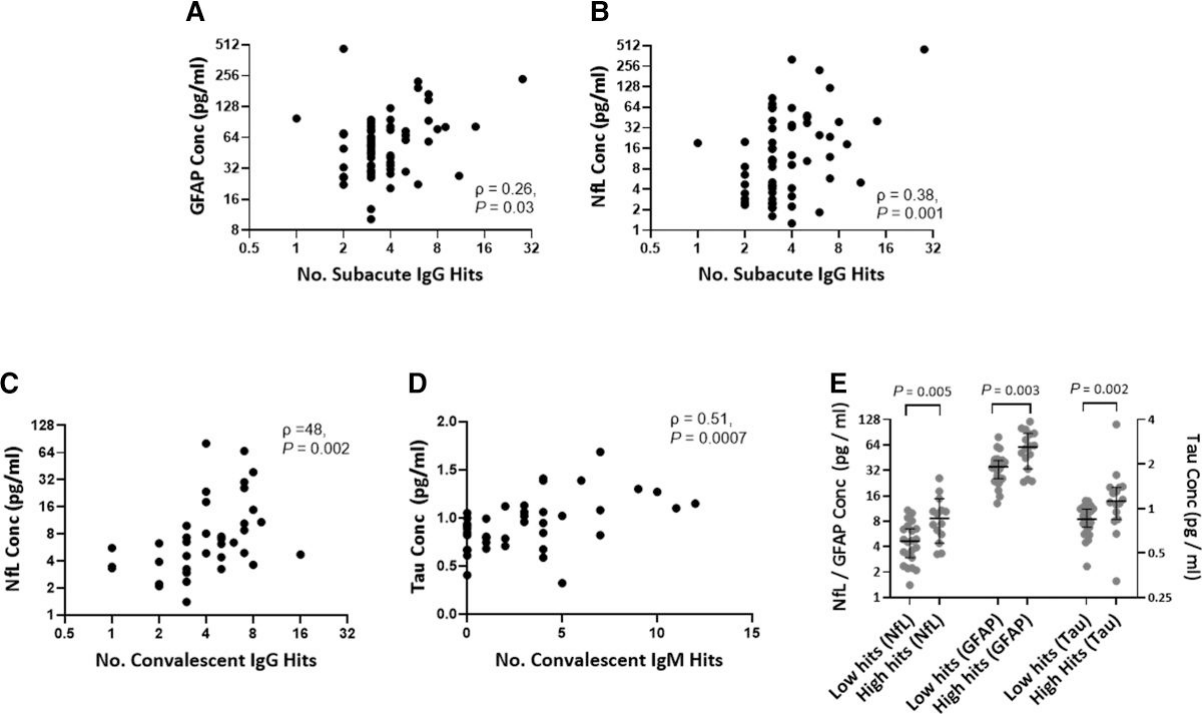
**Relationship between serum brain injury biomarkers and autoantibody profiles.** (**A** and **B**) Correlation between number of IgG hits and serum GFAP and NfL concentrations at the subacute time point. (**C**) Correlation between number of IgG hits and serum NfL concentrations at the convalescent time point. (**D**) Correlation between number of IgM hits and serum total tau concentrations at the convalescent time point. (**E**) Comparison of convalescent serum brain injury biomarker concentrations between patients with high IgM responses (>3 IgM hits Z > 3) versus those with low IgM responses (<3 IgM hits Z > 3). Two-group unpaired comparisons are by Mann-Whitney U-test, correlations are by Spearman’s rank.

To understand whether there was a relationship between inflammatory profiles and brain injury biomarkers, we compared brain injury biomarker levels with cytokines and autoantibody responses. At the subacute time point, serum GFAP and NfL concentrations positively correlated with both the number of IgG hits [GFAP and NfL versus IgG hits: ρ = 0.26, *P* = 0.03 and ρ = 0.38, *P* = 0.001, respectively (Fig. 3A and B)] and increased proinflammatory cytokine responses (GFAP and NfL versus pro-inflammatory load score ρ = 0.53, *P* < 0.0001 and ρ = 0.65, *P* < 0.0001, respectively), but there was no such relationship between serum total tau concentration and number of IgG hits or cytokine response (ρ = −0.02, *P* = 0.90 and ρ = −0.17, *P* = 0.2). The number of IgM hits also correlated with serum NfL concentration (ρ = 0.33, *P* = 0.006), but not with GFAP or total tau (ρ = 0.20, *P* = 0.10, and ρ = 0.07, *P* = 0.57, respectively). The relationship between brain injury biomarkers and the top 10 most frequently detected autoantibodies was investigated; after Bonferroni correction, serum NfL concentrations were associated with the Z-score of IgG autoantibodies against NfL, SFTPA1 and MYBPHL (ρ = 0.35, *P* = 0.002, ρ = 0.38, *P* = 0.001 and ρ = 0.41, *P* = 0.0005, respectively), but none of the top 10 autoantibodies retained significance against serum GFAP or total tau concentrations after correcting for multiple comparisons. Importantly, there was no suggestion that autoantibodies against brain antigens associated more strongly with brain injury biomarker concentrations than those targeting non-brain antigens. There was no association between serum biomarker concentrations and autoantibody profiles in the healthy control group.

In the convalescent period, the number of IgG hits once again correlated with serum NfL concentrations (ρ = 0.48, *P* = 0.002; Fig. 3C), but not GFAP or total tau (ρ = 0.12, *P* = 0.46, ρ = −0.08, *P* = 0.63, respectively). The relationship between brain injury biomarkers and cytokine profiles seen in the acute phase was replicated in convalescent patients, with elevations in proinflammatory cytokines (IL-1β, IL-6 and TNFα as described by pro-inflammatory load score) associating with raised NfL and GFAP, but not total tau (pro-inflammatory load score versus NfL: ρ = 0.55, *P* < 0.0001; GFAP: ρ = 0.26, *P* = 0.05; total tau ρ = 0.1, *P* = 0.43).

A comparable relationship between subacute brain injury biomarkers and pro-inflammatory cytokine concentrations was seen in the influenza cohort (e.g. TNFa versus NfL and GFAP: ρ = 0.56, *P* = 0.0001 and ρ = 0.60, *P* < 0.0001, respectively, and IL-6 versus NfL and GFAP: ρ = 0.35, *P* = 0.02 and ρ = 0.36, *P* = 0.02, respectively).

### IgM autoantibodies at convalescence are associated with brain injury biomarker elevation, notably tau

At the convalescent time point, however, there was an association between number of IgM hits and all brain injury biomarkers, particularly total tau (GFAP: ρ = 0.45, *P* = 0.004; NfL: ρ = 0.50, *P* = 0.001; total tau: ρ = 0.51, *P* = 0.0007; Fig. 3D). To investigate this relationship further, patients were dichotomized into either high IgM responder (≥3 IgM hits) versus low IgM responder (<3 IgM hits) groups, and the levels of brain-injury biomarkers compared. Serum concentrations of all three biomarkers were higher in the high IgM responder group, but again total tau was the most highly significant difference [GFAP: 58.2 (32.6–87.05) versus 37.8 (23.8–43.1), *P* = 0.03; NfL: 7.5 (5.2–16.5) versus 4.6 (3.0–8.1), *P* = 0.026; total tau: 1.1 (0.9–1.3) versus 0.8 (0.7–0.9), *P* = 0.001; Fig. 3E].

## Discussion

The aim of this study was to examine how frequently brain injury occurred in COVID-19, both acutely and in convalescence, and whether elevated brain injury biomarkers were associated with a dysregulated host inflammatory response. We demonstrated that brain injury biomarkers are elevated in a severity-dependent manner in the acute phase, and that these elevations are associated with both raised pro-inflammatory cytokines and the presence of autoantibodies. When patients were followed-up (∼4 months post-admission), there was evidence that this immunological dysregulation had not fully resolved and was associated with serum markers of ongoing active brain injury (namely NfL), albeit to a lesser degree than in the acute illness. In addition, in convalescent patients, there appeared to be a second, separate, process, which was characterized by a different pattern of serum brain injury biomarkers (more specifically elevation of total tau), which was not related to initial COVID-19 severity or pro-inflammatory cytokine levels but was associated with the presence of IgM autoantibodies. We observed autoantibody responses to many different targets (most commonly lung surfactant protein A1 and myelin associated glycoprotein), but the particular target of the autoantibody did not seem to relate to the presence of brain injury; rather, it seemed that the more diverse the autoantibody repertoire generated (reflecting a more generalized immune response), the more significant the degree of brain injury. It was notable that the presence of autoantibodies against brain antigens was no stronger predictor of brain injury than those targeting non-brain antigens, suggesting that the brain injury occurred in the setting of a general dysregulated immune response rather than as a result of directly pathogenic autoantibodies; this is further supported by the fact that the strength of signal generated by the autoantibodies was often significantly lower than that generated by the anti-spike and anti-nucleocapsid antibodies, which may suggest that the autoantibodies detected are low-affinity species, less likely to be directly pathogenic.

Our data confirm and extend previous studies investigating brain injury biomarkers in COVID-19, which have suggested that blood NfL concentrations are elevated in acute COVID-19 infection, and associate with severity of illness and therefore poor outcome.^5–15^ Whilst NfL and GFAP can be found in non-CNS tissue (peripheral nerve and gut, respectively), contemporaneously elevated concentrations of both is an established marker of CNS injury, with the brain representing the dominant source.^30,31^ A longitudinal cohort study by members in our collaboration, demonstrated that serum NfL and GFAP levels had returned to baseline by 6 months following admission,^7^ suggesting that the persistent elevation in NfL at 4 months in our cohort is capturing the end of this period of active brain injury. The late elevations in total tau seen in our cohort, however, are novel, as there is no precedent in the COVID-19 literature for this. Elevated serum total tau concentrations have been described in patients with tauopathies such as Alzheimer’s disease and frontotemporal dementia,^32^ and are associated with trajectory of cognitive decline in these conditions.^33,34^ Larger cohorts will be required to replicate our COVID-19 finding and accurately delineate the association between late elevated total tau and clinical outcome, however the lack of association between initial disease severity and subsequent total tau elevation is tantalizing given the neuropsychological sequelae that occurs in a substantial minority of people with even mild COVID-19.

It is well recognized that viral infections can trigger autoantibody production, both low-affinity polyreactive species, as well as higher affinity-specific species such as anti-cardiolipin antibodies.^35,36^ This phenomenon has been replicated in COVID-19, with a number of studies describing the presence of autoantibodies to a plethora of targets including ‘traditional’ rheumatological autoantibodies as well as less clinically established autoantibodies such as those targeting type 1 interferons.^37–42^ The role of these autoantibodies is largely unknown. Although they appear to occur more commonly in severe illness, they may simply represent an epiphenomenon of tissue damage (perhaps even a useful mechanism for debris clearance, a putative role of natural autoantibodies). However, it has been suggested that autoantibodies to certain targets (such as interferons) may predispose to severe disease,^43^ and it appears that immune-complex formation is a potent driver of secondary immune cell activation in COVID-19.^44^

The associations seen in our data between brain injury biomarkers and dysregulation of both innate and adaptive immune responses may represent inflammatory mechanisms that drive neurological injury. The well documented impact of immune modulatory treatments in preventing severe COVID-19 provides strong evidence that a substantial component of the acute pathophysiology of COVID-19 relates to a dysregulated host response, rather than damage caused directly by the virus. Our data suggest that brain injury occurring during acute COVID-19 may also result from similar mechanisms, and provide a plausible mechanistic basis for these manifestations, given the scant evidence to support direct viral invasion of the brain by SARS-CoV-2.^1^

Our data do not define causality between the immunological parameters and the presence of brain injury. In the acute phase, both may be influenced by additional factors that drive severe disease. Indeed, the immunological changes may be occurring in response to tissue injury, rather than causing it. However, given the growing evidence of the detrimental effects of excess inflammation in COVID-19 more broadly, it is plausible that the elevation of brain injury biomarkers is driven by a maladaptive host response.^45^ This may be the result of neuroinflammation *per se*,^46–49^ or inflammatory injury to the cerebrovascular bed, which subsequently results in microvascular ischaemic brain injury.^50–53^ Similar considerations may apply to the convalescent phase of illness, where the association of IgM autoantibodies with serum tau could represent a persisting immunological dyscrasia driving brain injury. The relative specificity of tau at this phase of the illness may represent tissue specificity of the process (tau is a dendritic and axonal marker).

Importantly, the data from our influenza control group suggest that the occurrence of brain injury in the acute phase of COVID-19 is not unique to this infection. In fact, a single small study also suggested that patients with bacterial pneumonia displayed higher blood markers of brain injury than patients with COVID-19,^9^ and therefore the processes described in our paper are likely to be relevant to severe infective illnesses more broadly. This being the case, data from COVID-19 studies may serve to help mitigate against the neurological sequelae of severe illness in the future.^54^

In conclusion, we have demonstrated that markers of brain injury are associated with dysregulated immunological responses in COVID-19, and that there may be a separate late process irrespective of initial disease severity which is characterized by elevated serum total tau concentrations and the presence of IgM autoantibodies.

## Supplementary Material

awac321_Supplementary_Data

## Appendix 1

### Cambridge NeuroCOVID Group

Fahim Anwar, Kieren Allinson, Junaid Bhatti, Edward T. Bullmore, Dorothy A. Chatfield, David Christmas, Alasdair J. Coles, Jonathan P. Coles, Marta Correia, Tilak Das, Paul C. Fletcher, Alasdair W. Jubb, Victoria C. Lupson, Anne E. Manktelow, David K. Menon, Andrew Michell, Edward J. Needham, Virginia F. J. Newcombe, Joanne G. Outtrim, Linda Pointon, Christopher T. Rodgers, James B. Rowe, Catarina Rua, Nyarie Sithole, Lennart R. B. Spindler, Emmanuel A. Stamatakis, Jonathan Taylor, Fernanda Valerio, Barry Widmer, Guy B. Williams, Patrick F. Chinnery.

### CITIID-NIHR COVID-19 BioResource Collaboration

John Allison, Gisele Alvio, Ali Ansaripour, Sharon Baker, Stephen Baker, Laura Bergamaschi, Areti Bermperi, Ariana Betancourt, Heather Biggs, Sze-How Bong, Georgie Bower, John R. Bradley, Karen Brookes, Ashlea Bucke, Ben Bullman, Katherine Bunclark, Helen Butcher, Sarah Caddy, Jo Calder, Laura Caller, Laura Canna, Daniela Caputo, Matt Chandler, Yasmin Chaudhry, Patrick Chinnery, Debbie Clapham-Riley, Daniel Cooper, Chiara Cossetti, Cherry Crucusio, Isabel Cruz, Martin Curran, Jerome D. Coudert, Eckart M. D. D. De Bie, Rnalie De Jesus, Aloka De Sa, Anne-Maree Dean, Katie Dempsey, Eleanor Dewhurst, Giovanni di Stefano, Jason Domingo, Gordon Dougan, Benjamin J. Dunmore, Anne Elmer, Madeline Epping, Codie Fahey, Stuart Fawke, Theresa Feltwell, Christian Fernandez, Stewart Fuller, Anita Furlong, Iliana Georgana, Anne George, Nick Gleadall, Ian G Goodfellow, Stefan Gräf, Barbara Graves, Jennifer Gray, Richard Grenfell, Ravindra K. Gupta, Grant Hall, William Hamilton, Julie Harris, Sabine Hein, Christoph Hess, Sarah Hewitt, Andrew Hinch, Josh Hodgson, Myra Hosmillo, Elaine Holmes, Charlotte Houldcroft, Christopher Huang, Oisín Huhn, Kelvin Hunter, Tasmin Ivers, Aminu Jahun, Sarah Jackson, Isobel Jarvis, Emma Jones, Heather Jones, Sherly Jose, Maša Josipović, Mary Kasanicki, Jane Kennet, Fahad Khokhar, Yvonne King, Nathalie Kingston, Jenny Kourampa, Emma Le Gresley, Elisa Laurenti, Ekaterina Legchenko, Paul J. Lehner, Daniel Lewis, Emily Li, Rachel Linger, Paul A. Lyons, Michael Mackay, John C. Marioni, Jimmy Marsden, Jennifer Martin, Cecilia Matara, Nicholas J. Matheson, Caroline McMahon, Anne Meadows, Sarah Meloy, Vivien Mendoza, Luke Meredith, Nicole Mende, Federica Mescia, Alice Michael, Alexei Moulton, Rachel Michel, Lucy Mwaura, Francesca Muldoon, Francesca Nice, Criona O’Brien, Charmain Ocaya, Ciara O’Donnell, Georgina Okecha, Ommar Omarjee, Nigel Ovington, Willem H. Owehand, Sofia Papadia, Roxana Paraschiv, Surendra Parmar, Ciro Pascuale, Caroline Patterson, Christopher Penkett, Marlyn Perales, Marianne Perera, Isabel Phelan, Malte Pinckert, Linda Pointon, Petra Polgarova, Gary Polwarth, Nicole Pond, Jane Price, Venkatesh Ranganath, Cherry Publico, Rebecca Rastall, Carla Ribeiro, Nathan Richoz, Veronika Romashova, Sabrina Rossi, Jane Rowlands, Valentina Ruffolo, Jennifer Sambrook, Caroline Saunders, Natalia Savinykh Yarkoni, Katherine Schon, Mayurun Selvan, Rahul Sharma, Joy Shih, Kenneth G. C. Smith, Sarah Spencer, Luca Stefanucci, Hannah Stark, Jonathan Stephens, Kathleen E Stirrups, Mateusz Strezlecki, Charlotte Summers, Rachel Sutcliffe, James E. D. Thaventhiran, Tobias Tilly, Zhen Tong, Hugo Tordesillas, Carmen Treacy, Mark Toshner, Paul Townsend, Carmen Treacy, Lori Turner, Phoebe Vargas, Bensi Vergese, Julie von Ziegenweidt, Neil Walker, Laura Watson, Jennifer Webster, Michael P. Weekes, Nicola K. Wilson, Jennifer Wood, Jieniean Worsley, Marta Wylot, Anna Yakovleva, Cissy Yong and Julie-Anne Zerrudo.

### NIHR Cambridge Clinical Research Facility

Caroline Saunders, Anne Elmer.
