# Correction to: Brain injury in COVID-19 is associated with dysregulated innate and adaptive immune responses

**DOI:** 10.1093/brain/awad371

**Published:** 2023-11-06

Edward J. Needham, Alexander L. Ren, Richard J. Digby, Emma J. Norton, Soraya Ebrahimi, Joanne G. Outtrim, Doris A. Chatfield, Anne E. Manktelow, Maya M. Leibowitz, Virginia F. J. Newcombe, Rainer Doffinger, Gabriela Barcenas-Morales, Claudia Fonseca, Michael J. Taussig, Rowan M. Burnstein, Romit J. Samanta, Cordelia Dunai, Nyarie Sithole, Nicholas J. Ashton, Henrik Zetterberg, Magnus Gisslén, Arvid Edén, Emelie Marklund, Peter J. M. Openshaw, Jake Dunning, Michael J. Griffiths, Jonathan Cavanagh, Gerome Breen, Sarosh R. Irani, Anne Elmer, Nathalie Kingston, Charlotte Summers, John R. Bradley, Leonie S. Taams, Benedict D. Michael, Edward T. Bullmore, Kenneth G. C. Smith, Paul A. Lyons, Alasdair J. Coles, David K. Menon, Cambridge NeuroCOVID Group, CITIID-NIHR COVID-19 BioResource Collaboration, Cambridge NIHR Clinical Research Facility. Brain injury in COVID-19 is associated with dysregulated innate and adaptive immune responses. *Brain*. 2022;145:4097–4107. https://doi.org/10.1093/brain/awac321

In the original article, the name of the author Arvid Edén was published incorrectly as Arden Edén. This has been corrected in the online version of the article.

