# Supplementary material for: Brain injury in COVID-19 is associated with dysregulated innate and adaptive immune responses
Source: Brain. 2022 Sep 6;145(11):4097–107. doi: 10.1093/brain/awac321 (PMC9494359; doi:10.1093/brain/awac321)
Supplement: awac321_Supplementary_Data [file awac321_supplementary_data.pdf]

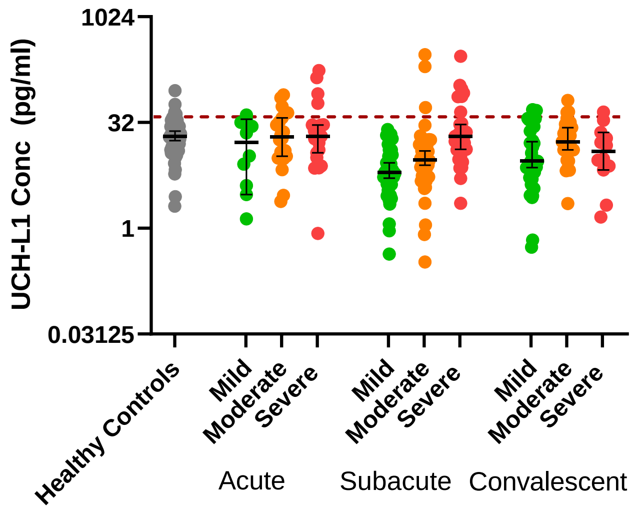

Supplementary Figure 1. UCH-L1 concentrations in healthy controls and COVID-19 patients. Most datapoints fall below the assay's functional lower limit of quantification (denoted by maroon dashed line), and therefore these data were not included in the analysis.

Central Nervous System

ANXA4 APP BSG CDH13 CDR2 CHRNA9 COL4A3BP DCN DPYSL5 DRD2 ELAVL4 GABBR1 GABRA1 GABRB3 GAD1 GAD2 GFAP GLRA1 GRIA3 GRIA4 GRIN1 GRIN2A GRIN3A GRIN3B GRINA GRM1 GRM2 GRM3 GRM4 GRM7 GRM8 KCNJ10 LGI1 MAG MAPT MBP MOG NEFL NOVA1 OMG PNMA1 PNMA2 S100B SNCA SSB TPH1 TROVE2 TUBB3 UCHL1 ZIC4

Kidney

AGTR1 COL4a3 GSTT1 MPO NPHS2 PLA2R PRTN3 SLC22A12 TMEM174 UMOD VIM

Lung

AGER COL1A1 COL1A2 COL5A2 SCGB1A1 SCGB3A2 SFTPA1 SFTPA2 SFTPC TUBA1B

Muscle

ACTA1 ANKRD23 CHRNA10 DUPD1 HARS IDI2 PPP1R27 TPM1 TTN UCP3

Heart

CHRM2 EDNRA LRRC10 MYBPHL MYL4 MYL7 NPPA NPPB TNNI3 TNNT2

HLA

CD74 HLA-A HLA-B HLA-C HLA-DMA HLA-DMB HLA-DOA HLA-DOB HLA-DPA1 HLA-DPB1 HLA-DQA1 HLA-DQB1 HLA-DQB2 HLA-DRA HLA-DRB1 HLA-DRB3 HLA-DRB4 HLA-DRB5 HLA-E HLA-F HLA-G

Endocrine

FSHB GHRHR GNRHR POMC PRL TSHB TPO TSHR

Blood Brain Barrier

CLDN5 LAMC2 SELE SLC2A1 TJP1

Coagulation

ADAMTS13 ANXA5 APOH F2 F7 F8 F9 FGB PROC PROS1

Ubiquitous

ACE CDH1 CEACAM1 CEACAM5 CENPB CENPH DBT DDC DLAT IFNA1 KRT18 NUP210 TGM2 ZNF397

COVID-19

Spike protein

Nucleocapsid

Supplementary Figure 2. Protein microarray antigen composition. Antigens are grouped by the tissue where they are predominantly expressed (based on GTEx RNAseq data [www.gtexportal.org]) ; it should be noted that expression is likely to also occur in other tissues to varying degrees.

A

	Cambridge Cohort	Gothenburg Cohort	Influenza Cohort	Healthy Controls	All Cambridge Admissions	All Gothenburg Admissions
n =	122	53	45	59	1666	137
Age (Median [IQR])	50 (31-62)	51 (35-59)	44 9 30-50)	50 (32-62)	67 (51-78)	56 (38-69)
Sex (No Male [%])	60 [49]	33 [62]	23 (51)	21 [35]	899 (54)	78 (57)
Severity (n = [%])						
Mild	70 (57)	0 (0)	22 (49)	-	NK	NK
Moderate	31 (25)	41 (77)	15 (33)	-	NK	NK
Severe	21 (17)	12 (23)	8 (18)	-	NK	NK

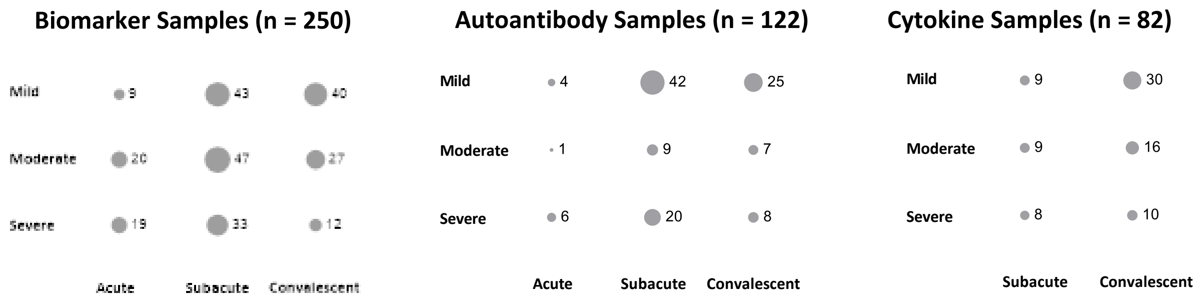

Supplementary Figure 3. (**A**) Demographic and (**B**) sample details for biomarker quantification, autoantibody and cytokine profiling experiments. All Cambridge and All Gothenburg columns relate to all patients admitted with COVID-19 to these hospitals during the recruitment period. *NK = not known.*

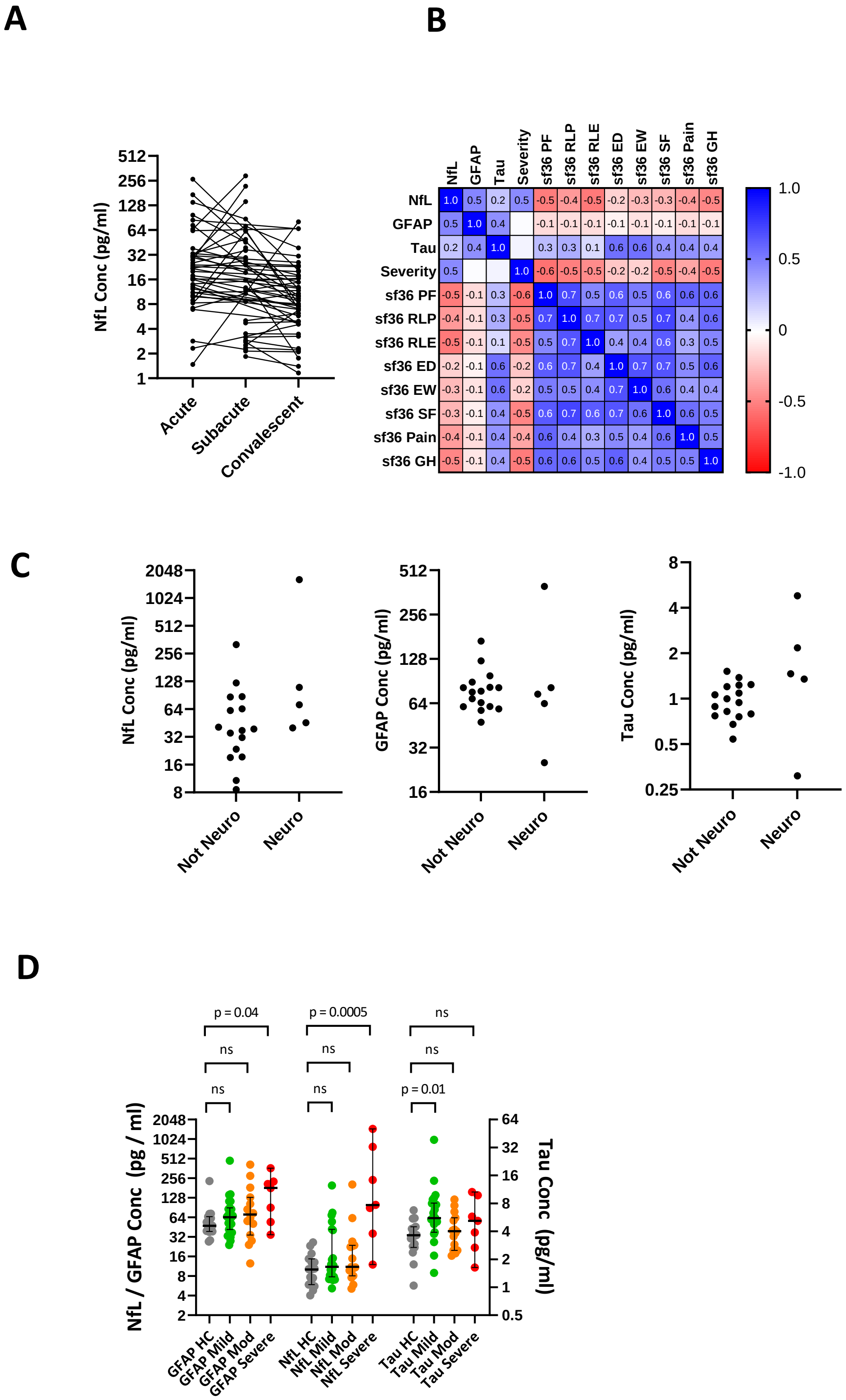

Supplementary Figure 4. A) Spaghetti plot displaying temporal profiles of NfL in patients who contributed longitudinal samples **B**) Correlation matrix of brain injury biomarkers and SF36 quality of

life measure components **C**) Comparison of brain injury biomarkers between those patients with severe COIVD-19 who developed syndromic neurological diagnoses (mononeuritis multiplex n = 3, opsoclonus myoclonus n = 1, peripheral neuropathy with concurrent encephalopathy n = 1) versus those who did not. **D**) Plasma concentrations of brain-injury biomarkers in patients admitted with influenza.

A

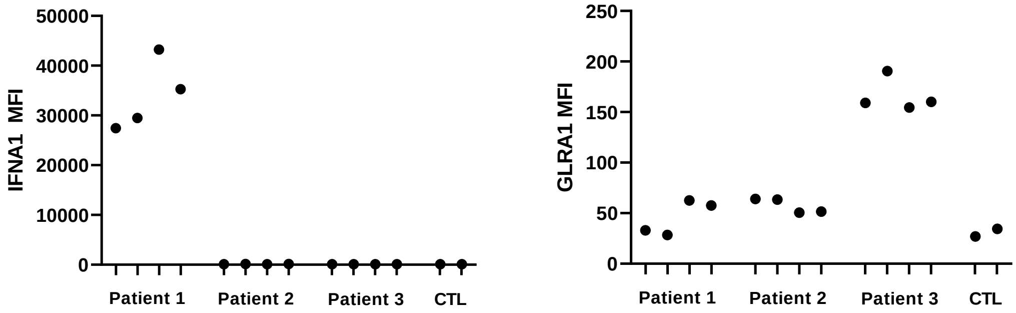

B

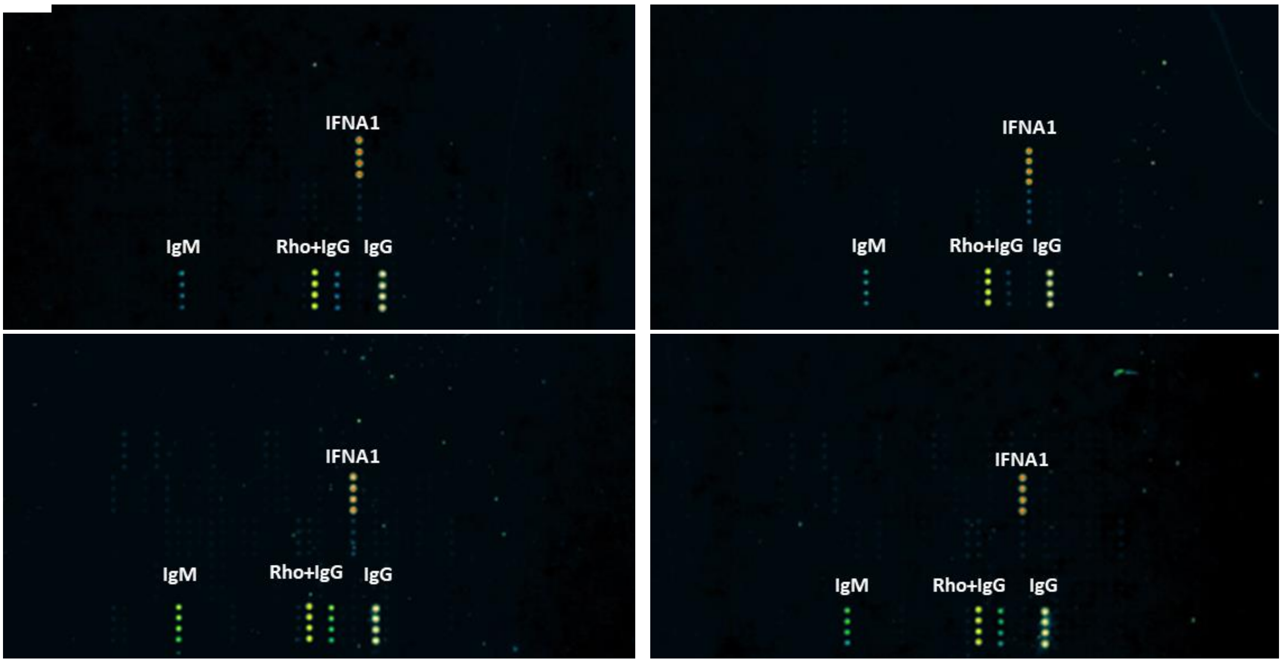

Supplemental Figure 5. A) Technical replicates of autoantibody profiling for 3 patients, one with a strong response to interferon alpha (patient 1), and one with a weaker response to glycine receptor alpha 1 (patient 3). *CTL = subunits incubated with reagents but no serum* **B**) Photographs of the strong response against interferon alpha. *IgG, Rho+IgG and IgM are positive control spots.*

	Acute (n = 48)	Subacute (n = 123)	Convalescent (n = 79)
**GFAP HC**	42.2 [32.2 - 68.6]	42.2 [32.2 - 68.6]	42.2 [32.2 - 68.6]
**GFAP Mild**	55.3 [43.7-113]	34.5 [27.5 - 70.5]	42.15 [26.2 - 79.2]
**GFAP Mod**	143.5 [64.6-259]	54.3 [40.7 - 80.7]	55.8 [44.9 - 104.0]
**GFAP Severe**	98.1 [87.3 - 208.5]	82.2 [64 - 165.9]	41.9 [34.5 - 74.6]
**NfL HC**	5.5 [3.6 - 10.4]	5.5 [3.6 - 10.4]	5.5 [3.6 - 10.4]
**NfL Mild**	8.2 [2.6 - 15.9]	4.2 [2.8-9.1]	4.8 [3.0 - 10.0]
**NfL Mod**	19.2 [11.1 - 30.7]	10.1 [5.1 - 16.0]	10.4 [6.9 - 18.0]
**NfL Severe**	30.6 [14.3 - 81.3]	45.6 [30.8-98.7]	10.8 [7.4 - 19.4
**Tau HC**	0.72 [0.60 - 1.04]	0.72 [0.60 - 1.04]	0.72 [0.60 - 1.04]
**Tau Mild**	0.78 [0.65 - 1.21]	0.80 [0.66 - 1.03]	0.95 [0.75 - 1.11]
**Tau Mod**	0.74 [0.49 - 1.10]	0.39 [0.18 - 0.73]	0.90 [0.71 - 1.27]
**Tau Severe**	0.68 [0.33 - 1.02]	0.94 [0.68 - 1.24]	0.91 [0.73 - 1.27]

Supplementary Table 1. Brain injury biomarker data. Values shown are median [IQR].

HC = healthy controls (n = 59); Mild (n = 70), Mod (Moderate; n = 72), and Severe (n = 33) relates to severity of COVID-19.

Frequency of Positive Autoantibody Hits (Z ≥ 3)

IgG			IgM		
Name	*% Controls Z ≥ 3*	*% COVID Z ≥ 3*	*Name*	*% Controls Z ≥ 3*	*% COVID Z ≥ 3*
ACE	2.6	0.8	ACE	0.0	5.6
ACTA1	2.6	0.8	ACTA1	2.6	0.8
ADAMTS13	2.6	0.0	ADAMTS13	1.3	0.0
AGER	0.0	0.0	AGER	1.3	2.4
AGTR1	1.3	0.0	AGTR1	2.6	0.0
ANKRD23	2.6	0.0	ANKRD23	0.0	0.0
ANXA4	1.3	0.0	ANXA4	2.6	1.6
ANXA5	1.3	0.0	ANXA5	1.3	1.6
APOH	1.3	0.0	APOH	2.6	0.8
APP	1.3	0.0	APP	1.3	0.0
BSG	2.6	0.0	BSG	1.3	6.4
CD74	1.3	0.0	CD74	1.3	5.6
CDH1	1.3	0.8	CDH1	2.6	0.8
CDH13	1.3	0.0	CDH13	1.3	4.0
CDR2	2.6	0.0	CDR2	2.6	0.0
CEACAM1	1.3	0.0	CEACAM1	0.0	3.2
CEACAM5	2.6	0.0	CEACAM5	1.3	0.8
CENPB	1.3	0.0	CENPB	2.6	0.8
CENPH	3.9	0.8	CENPH	0.0	1.6
CHRM2	1.3	0.8	CHRM2	0.0	1.6
CHRNA10	1.3	0.8	CHRNA10	1.3	7.2
CHRNA9	0.0	1.6	CHRNA9	0.0	5.6
CLDN5	0.0	2.4	CLDN5	0.0	0.8
COL1A1	2.6	3.2	COL1A1	1.3	0.0
COL1A2	5.2	0.8	COL1A2	2.6	3.2
COL4a3	2.6	0.8	COL4a3	2.6	1.6
COL4A3BP	2.6	0.8	COL4A3BP	2.6	1.6
COL5A2	2.6	4.8	COL5A2	1.3	1.6
DBT	1.3	1.6	DBT	1.3	1.6
DCN	1.3	0.8	DCN	1.3	2.4
DDC	0.0	0.8	DDC	1.3	4.8
DLAT	2.6	6.4	DLAT	2.6	2.4
DPYSL5	1.3	0.8	DPYSL5	1.3	0.8
DRD2	1.3	0.0	DRD2	1.3	0.8
DUPD1	0.0	6.4	DUPD1	2.6	4.8
EDNRA	1.3	5.6	EDNRA	1.3	0.8
ELAVL4	2.6	2.4	ELAVL4	1.3	0.0
F2	1.3	0.8	F2	1.3	0.0
F7	1.3	0.0	F7	1.3	0.0
F8	1.3	4.0	F8	2.6	3.2
F9	1.3	2.4	F9	1.3	0.0
FGB	0.0	4.8	FGB	0.0	1.6
FSHB	3.9	0.0	FSHB	1.3	0.0
GABBR1	1.3	5.6	GABBR1	2.6	6.4
GABRA1	1.3	8.8	GABRA1	1.3	0.8
GABRB3	0.0	3.2	GABRB3	1.3	2.4
GAD1	2.6	1.6	GAD1	2.6	0.8
GAD2	1.3	0.8	GAD2	3.9	1.6
GFAP	2.6	3.2	GFAP	2.6	0.8
GHRHR	0.0	5.6	GHRHR	1.3	7.2
GLRA1	1.3	5.6	GLRA1	1.3	4.8
GNRHR	1.3	3.2	GNRHR	0.0	3.2
GRIA2	1.3	0.8	GRIA2	1.3	0.8
GRIA3	1.3	0.8	GRIA3	1.3	4.8
GRIA4	1.3	0.0	GRIA4	2.6	0.0
GRIN1	2.6	2.4	GRIN1	0.0	1.6
GRIN2A	0.0	2.4	GRIN2A	2.6	4.0
GRIN3A	1.3	0.0	GRIN3A	1.3	0.8
GRIN3B	0.0	1.6	GRIN3B	1.3	2.4
GRINA	1.3	3.2	GRINA	1.3	4.8
GRM1	1.3	0.0	GRM1	0.0	0.8
GRM2	3.9	0.8	GRM2	0.0	0.8
GRM3	1.3	0.0	GRM3	1.3	0.0
GRM4	0.0	0.8	GRM4	0.0	0.0
GRM7	2.6	0.0	GRM7	0.0	0.0
GRM8	0.0	0.8	GRM8	1.3	0.8
GSTT1	1.3	0.8	GSTT1	1.3	3.2
HARS	2.6	1.6	HARS	2.6	4.0
HLA-A	1.3	0.8	HLA-A	1.3	0.8
HLA-B	1.3	0.8	HLA-B	1.3	1.6
HLA-C	1.3	0.8	HLA-C	1.3	0.0
HLA-DMA	2.6	1.6	HLA-DMA	1.3	0.8
HLA-DMB	2.6	1.6	HLA-DMB	2.6	0.0
HLA-DOA	2.6	0.0	HLA-DOA	1.3	2.4
HLA-DOB	1.3	0.0	HLA-DOB	3.9	0.8
HLA-DPA1	2.6	0.0	HLA-DPA1	1.3	2.4
HLA-DPB1	3.9	0.0	HLA-DPB1	1.3	0.8

HLA-DQA1	2.6	0.0		HLA-DQA1	1.3	0.8
HLA-DQB1	5.2	0.0		HLA-DQB1	0.0	0.8
HLA-DQB2	1.3	0.0		HLA-DQB2	1.3	0.0
HLA-DRA	0.0	0.0		HLA-DRA	0.0	5.6
HLA-DRB1	1.3	1.6		HLA-DRB1	0.0	7.2
HLA-DRB3	2.6	0.0		HLA-DRB3	0.0	0.8
HLA-DRB4	3.9	2.4		HLA-DRB4	0.0	6.4
HLA-DRB5	2.6	0.0		HLA-DRB5	1.3	0.0
HLA-E	0.0	0.0		HLA-E	0.0	0.8
HLA-F	1.3	0.8		HLA-F	0.0	0.0
HLA-G	1.3	1.6		HLA-G	1.3	2.4
IDI2	2.6	0.0		IDI2	0.0	1.6
IFNA1	1.3	0.8		IFNA1	1.3	0.0
KCNJ10	2.6	4.0		KCNJ10	1.3	0.0
KRT18	1.3	8.0		KRT18	1.3	3.2
LAMC2	1.3	0.8		LAMC2	1.3	3.2
LGI1	1.3	8.0		LGI1	1.3	6.4
LRRC10	2.6	0.0		LRRC10	1.3	0.0
MAG	0.0	9.6		MAG	1.3	4.0
MAPT	2.6	0.0		MAPT	1.3	0.8
MBP	1.3	0.0		MBP	1.3	2.4
MOG	1.3	0.0		MOG	1.3	0.8
MPO	2.6	0.8		MPO	1.3	6.4
MYBPHL	0.0	4.8		MYBPHL	1.3	9.6
MYL4	1.3	0.0		MYL4	1.3	1.6
MYL7	1.3	0.0		MYL7	0.0	0.0
NEFL	2.6	2.4		NEFL	0.0	5.6
NOVA1	2.6	0.0		NOVA1	2.6	5.6
NPHS2	2.6	3.2		NPHS2	2.6	2.4
NPPA	1.3	0.0		NPPA	3.9	0.0
NPPB	2.6	4.0		NPPB	0.0	2.4
Nucleocapsid protei	1.3	59.2		Nucleocapsid protei	3.9	0.0
NUP210	2.6	0.8		NUP210	2.6	1.6
OMG	1.3	0.0		OMG	0.0	0.0
PLA2R1	1.3	4.0		PLA2R1	1.3	0.0
PNMA1	1.3	0.0		PNMA1	2.6	3.2
PNMA2	2.6	2.4		PNMA2	1.3	0.0
POMC	1.3	0.0		POMC	1.3	0.0
PPP1R27	2.6	0.0		PPP1R27	1.3	2.4
PRL	2.6	0.0		PRL	1.3	0.8
PROC	1.3	2.4		PROC	1.3	0.8
PROS1	1.3	0.0		PROS1	2.6	0.8
PRTN3	2.6	0.8		PRTN3	2.6	0.8
Rhodamine+IgG647	1.3	0.8		Rhodamine+IgG647	1.3	0.0
S100B	1.3	0.0		S100B	1.3	0.0
SCGB1A1	1.3	0.0		SCGB1A1	1.3	0.0
SCGB3A2	1.3	0.0		SCGB3A2	1.3	2.4
SELE	1.3	1.6		SELE	1.3	4.0
SFTPA1	0.0	8.8		SFTPA1	1.3	3.2
SFTPA2	1.3	0.8		SFTPA2	2.6	0.0
SFTPC	3.9	1.6		SFTPC	0.0	4.0
SLC22A12	2.6	0.0		SLC22A12	0.0	0.0
SLC2A1	0.0	4.8		SLC2A1	1.3	14.4
SNCA	0.0	0.0		SNCA	1.3	0.0
Spike protein	1.3	80.0		Spike protein	1.3	36.0
SSB	1.3	3.2		SSB	2.6	6.4
TGM2	1.3	0.0		TGM2	2.6	0.8
TJP1	1.3	0.0		TJP1	1.3	1.6
TMEM174	1.3	2.4		TMEM174	0.0	5.6
TNNI3	2.6	2.4		TNNI3	1.3	3.2
TNNT2	2.6	0.8		TNNT2	1.3	0.8
TPH1	1.3	1.6		TPH1	1.3	3.2
TPM1	3.9	0.0		TPM1	1.3	0.8
TPO	1.3	1.6		TPO	1.3	1.6
TROVE2	2.6	4.8		TROVE2	1.3	0.8
TSHB	1.3	0.0		TSHB	1.3	0.0
TSHR	1.3	2.4		TSHR	0.0	0.8
TTN	1.3	0.0		TTN	2.6	2.4
TUBA1B	2.6	0.0		TUBA1B	1.3	0.0
TUBB3	1.3	0.0		TUBB3	1.3	3.2
UCHL1	1.3	0.0		UCHL1	1.3	2.4
UCP3	1.3	1.6		UCP3	2.6	0.0
UMOD	1.3	4.0		UMOD	3.9	1.6
VIM	2.6	0.0		VIM	2.6	1.6
ZIC4	1.3	0.0		ZIC4	0.0	0.8
ZNF397	2.6	1.6		ZNF397	1.3	0.0

Supplementary Table 2 Frequency of Positive Autoantibody hits
